# Identification and expression analysis of intergenic long non-coding RNAs (lincRNA) associated with stripe rust resistance in wheat

**DOI:** 10.3389/fpls.2026.1998911

**Published:** 2026-10-21

**Authors:** Xiaole Fang, Xiaoyu Wang, Tian Yang, Youning Wang, Xue Ao, Peina Cao, Pan Gong, Dongfang Ma

**Affiliations:** 1 MARA Key Laboratory of Sustainable Crop Production in the Middle Reaches of the Yangtze River (Co-Construction by Ministry and Province)/Hubei Key Laboratory of Waterlogging Disaster and Agricultural Use of Wetland/Interdisciplinary Innovation Research Center for Fungi and Mycosis Diagnosis and Therapy, College of Agriculture, Yangtze University, Jingzhou, China; 2 Hubei Key Laboratory of Resource Utilization and Quality Control of Characteristic Crops, College of Life Science and Technology, Hubei Engineering University, Xiaogan, China; 3 State Key Laboratory for Crop Stress Resistance and High-Efficiency Production, College of Plant Protection, Northwest A&F University, Yangling, China

**Keywords:** disease resistance, intergenic long non-coding RNA, stripe rust, transcriptome analysis, wheat

## Abstract

**Introduction:**

Long intergenic non-coding RNAs (lincRNAs) have emerged as important regulators of plant immune responses, yet their functional landscape in wheat (*Triticum aestivum*) during stripe rust (*Puccinia striiformis f. sp. tritici*) infection remains largely unexplored.

**Methods:**

In this study, a genome-wide identification of lincRNAs was performed using strand-specific RNA-seq data from wheat leaves following Pst inoculation. Functional enrichment, co-expression, lincRNA–miRNA–mRNA network prediction, and AlphaFold-based protein–RNA structure prediction were used to characterize candidate lincRNAs.

**Results:**

A total of 2,736 expressed lincRNAs were identified, of which 515 were differentially expressed during infection. Functional enrichment of genes located within 100 kb of the differentially expressed lincRNAs revealed significant associations with defense-related pathways, including MAPK signaling and plant–pathogen interaction. Coordinated expression patterns between lincRNAs and adjacent defense-related mRNAs suggested putative cis-regulatory associations. Furthermore, lincRNA–miRNA–mRNA network prediction identified 65 differentially expressed lincRNAs as potential endogenous target mimics for 21 conserved miRNAs, revealing a widespread competing endogenous RNA (ceRNA) regulatory network. Importantly, AlphaFold-based protein–RNA structure prediction demonstrated that the co-expressed lincRNA MSTRG.56980 forms a stable complex with the RGA1-type resistance protein TraesCS6B03G0179100.

**Discussion:**

This provides a first computational prediction of a possible physical interaction between a wheat lincRNA and an immune receptor. These integrative findings substantially expand the current understanding of non-coding regulatory networks in wheat immunity and provide a foundational resource for prioritizing candidate lincRNAs as targets for enhancing stripe rust resistance.

## 1 Introduction

As sessile organisms, plants must constantly adapt to their heterogeneous and fluctuating environment to ensure their growth, survival, and reproduction. Plants have evolved several mechanisms that allow them to recognize stress, integrate information, and modulate the expression of multiple genes to respond to new environmental conditions. Among the various molecular mechanisms underlying this adaptive plasticity, one of the most intriguing areas of study focuses on the role of non-coding RNA molecules in mediating environmental responses (Chorostecki et al., 2023). Long non-coding RNAs (lncRNAs) generally originate from polymerase II transcription but have distinct regulatory features, they usually exhibit limited protein-coding potential and modest expression levels (Yang et al., 2023). According to their genomic location relative to nearby protein-coding genes, lncRNAs can be broadly classified as lincRNAs, natural antisense transcripts (NATs), intronic non-coding RNAs (incRNAs) and sense lncRNAs (Ma et al., 2013).

LincRNAs which are transcribed from non-coding regions situated between protein-coding genes, have recently been recognized as key modulators of gene expression during plant immune responses (Shuai et al., 2014; Chen and Zhu, 2022). Although they do not encode proteins, lincRNAs fulfill diverse regulatory functions, including chromatin remodeling, transcriptional interference, and post-transcriptional control often mediated by their interaction with messenger RNAs or protein complexes (Deniz and Erman, 2017). Functional analyses in model species such as *Arabidopsis thaliana* and *Oryza sativa* have revealed that specific lincRNAs govern the expression of defense-associated genes under pathogen attack (Lu et al., 2017). In *Arabidopsis*, the lincRNA ELENA1 confers enhanced resistance to *Pseudomonas syringae* by interacting with mediator complex subunits to promote the transcriptional activation of PR1 (Seo et al., 2017). Similarly, in rice, ALEX1 stimulates jasmonate-mediated immunity, strengthening resistance against *Xanthomonas oryzae* pv. *oryzae* (Lei et al., 2025). Additional studies have highlighted the regulatory breadth of these molecules. In *Medicago truncatula*, the cold-inducible lincRNA MtCIR1 shows rapid upregulation within two hours of cold exposure, preceding the activation of adjacent MtCBF genes (Zhao et al., 2020). Certain lncRNAs have also been predicted to act as endogenous target mimics (eTMs), for instance, lncNAT159 is thought to sequester miR164, while lincRNA340 may similarly bind miR169 (Li et al., 2021). LincRNAs can influence gene expression either in cis or in trans by modulating mRNA stability, splicing patterns, chromatin accessibility, or translational efficiency (Yang et al., 2014). In rice ELENA1 enhances immune function by physically associating with OsWRKY7, a transcriptional repressor of defense-related genes (Cao et al., 2022a, b). Maize stress-responsive lincRNAs originating from transposable elements serve as regulatory nodes integrating both abiotic and biotic stress signals (Lv et al., 2019). Additional work has documented lincRNAs specifically induced in wheat during stripe rust infection (Cao et al., 2021). These studies demonstrate that lincRNAs are not transcriptional byproducts but integral components of plant immune networks.

Wheat is an important crop severely threatened by *Pst* (Han et al., 2025), the functional landscape of lincRNAs remains largely unresolved (Schwessinger, 2017). Although the wheat-*Pst* pathosystem is a well-established model for studying host-pathogen co-evolution, most research has focused on protein-coding components of immunity, particularly nucleotide-binding leucine-rich repeat (NLR) receptors and pathogenesis-related (PR) proteins (Jin et al., 2026). Recent transcriptome studies have reported that numerous wheat lncRNAs respond to *Pst* infection (Zhang et al., 2019), yet their precise classification as lincRNAs, their regulatory modes, and their mechanistic connections to mRNA networks remain poorly characterized (Song et al., 2022). This knowledge gap is especially critical given the rapid evolution of *Pst*, driven by frequent sexual recombination on *Berberis* species, which accelerates the emergence of virulent races capable of overcoming host resistance (Wang et al., 2017). While functional lincRNAs such as DRIR in *Arabidopsis* have been shown to regulate abiotic stress tolerance (Qin et al., 2017), comparable mechanistic insights in wheat-pathogen interactions are fragmented and incomplete.

The ceRNA hypothesis has emerged as a key regulatory paradigm in plants (Jin et al., 2023, 2024), wherein lincRNAs act as eTMs that sequester miRNAs to modulate mRNA expression (Bao et al., 2025). Meanwhile, deep learning-based tools such as AlphaFold have enabled the prediction of three-dimensional interactions between non-coding RNAs and their target proteins. However, neither ceRNA networks nor structural interactions of lincRNAs have been systematically explored in the wheat-*Pst* pathosystem.

To address this gap, this study aims to conduct a genome-wide identification of lincRNAs in wheat leaves during stripe rust infection and investigates their potential regulatory coordination with mRNAs. The study investigated whether lincRNA-mRNA crosstalk contributes to fungal immunity. Genome-guided transcriptome assembly was conducted to identify intergenic transcripts lacking coding potential, followed by co-expression network analysis to predict putative lincRNA-mRNA regulatory pairs. Expression dynamics was performed to assess genomic co-localization between differentially expressed lincRNAs and nearby mRNAs, particularly those encoding PR proteins or transporters, and lincRNA-mRNA pairs are validated through qRT-PCR. Our findings provide the first comprehensive view of lincRNA-mediated regulatory layers in the wheat-*Pst* pathosystem and establish a foundation for functional validation of candidate lincRNAs in disease resistance breeding.

## 2 Materials and methods

### 2.1 RNA sequence data retrieval

RNA-seq datasets used in this study were obtained from the National Center for Biotechnology Information Sequence Read Archive (NCBI SRA; accession PRJNA975982). The dataset contains transcriptomic profiles from leaves of the wheat cultivars MingXian 169 and ZhongSi following inoculation with *Pst*. Leaf tissues were sampled at 0 h, 24 h, 48 h, and 120 h, with three independent biological replicates at each time point. Although the original project included both cultivars, the present study focused exclusively on MingXian 169, a widely cultivated and *Pst*-susceptible wheat variety, to enable a clearer assessment of lincRNA responses during compatible interactions.

### 2.2 RNA sequence reads mapping and transcriptome assembly

Raw SRA files were converted to FASTQ format using Fastq-dump (v3.0.8), and read quality was assessed with FastQC (v0.12.1). Adapter sequences and low-quality bases were removed using Trim Galore (v0.6.10) to obtain high-quality clean reads. A splice-aware reference index of the Triticum aestivum genome (IWGSC RefSeq v2.1) was constructed using HISAT2 (v2.2.1), and the filtered reads were aligned to the reference genome. Alignment outputs were generated as SAM files and subsequently converted to sorted BAM files using Samtools (v1.13). Transcriptome assembly and expression quantification were performed with StringTie (v2.1.4) in genome-guided mode to reconstruct transcript structures and estimate expression levels. Normalized read counts and FPKM values were then calculated using Ballgown.

### 2.3 Bioinformatics pipeline for identifying lincRNAs

Transcript annotation refinement was performed using Gffcompare (v0.12.6) with the reference annotation provided to StringTie via the -r parameter. Only transcripts assigned the “u” class code, indicating intergenic loci, were retained. To reduce transcriptional noise, single-exon transcripts shorter than 200 bp were removed. Coding potential was evaluated using CPC2 (v2.0), CNCI (v2.0), and PLEK (v1.2). Only transcripts consistently classified as non-coding by all three tools were retained. Additional filtering excluded transcripts containing open reading frames (ORFs) longer than 300 nucleotides to ensure removal of potential protein-coding sequences. The remaining transcripts were translated in all six reading frames using EMBOSS Transeq, and the resulting peptide sequences were screened for conserved protein domains using HMMER against the Pfam database (E-value < 1e-5). Homology searches were also performed against the NCBI NR protein database using BLASTX (E-value < 1e-5), and any transcript with significant similarity to known proteins was discarded. Finally, expression support was assessed by retaining only transcripts with FPKM > 0.1 in at least one biological replicate.

### 2.4 Differential expression analysis and prediction of lincRNA-miRNA-mRNA networks

Differential expression of lincRNAs was assessed using DESeq2 (v1.38.3) on the OmicShare platform (https://www.omicshare.com/). Raw read counts for each lincRNA were modeled using a negative binomial generalized linear model. Statistical significance was determined using the Benjamini-Hochberg procedure to control the false discovery rate. LincRNAs were classified as differentially expressed if they met both thresholds: adjusted p-value ≤ 0.05 and |log_2_ (fold change)| ≥ 2. Mature wheat miRNA sequences were retrieved from miRBase, and potential interactions between lincRNAs, miRNAs, and mRNAs were predicted using the psRNATarget platform. Based on these predicted interactions, a lincRNA-miRNA-mRNA regulatory network was constructed using Cytoscape.

### 2.5 Target gene prediction and functional annotation of adjacent genes

To investigate potential cis-regulatory roles of the lincRNAs, protein-coding genes located within a 100-kb genomic window of each differentially expressed lincRNA were identified. Gene Ontology (GO) and Kyoto Encyclopedia of Genes and Genomes (KEGG) analysis of the identified mRNAs were performed on the OmicShare platform using Fisher’s exact test with Benjamini–Hochberg correction to control the false discovery rate (FDR < 0.05).

### 2.6 lincRNA-miRNA-mRNA network

To predict potential post-transcriptional regulatory interactions, the sequences of candidate lincRNAs and their cis-regulated adjacent mRNAs were submitted to the psRNATarget webserver (Dai et al., 2018) for miRNA target prediction. The analysis was performed using default parameters with the Expectation score threshold set to ≤ 4.5, following the recommended settings for plant miRNA target prediction. Predicted miRNA-target pairs were then filtered to retain only those involving differentially expressed lincRNAs and mRNAs. Based on these interactions, a lincRNA-miRNA-mRNA regulatory network was constructed and visualized using Cytoscape (v3.9.1).

### 2.7 Plant growth conditions and fungi treatment

Wheat cultivar Yangmai 158 seeds were surface-sterilized with 1% hydrogen peroxide and thoroughly rinsed with distilled water. They were then germinated on three layers of filter paper saturated with water at 25°C. Growth conditions were maintained at 25°C with a 16-h light/8-h dark photoperiod. Puccinia striiformis strain was cultured on PDA plates at 25°C for 3 days. Actively growing mycelial agar plugs (5 mm in diameter) were transferred to 150 mL conical flasks containing 50 mL of liquid mung bean broth and incubated at 25°C with shaking at 150 rpm for 3 days to produce conidia. At the two-leaf stage, the leaves were inoculated with the spore suspension (1×10^6^ spores/mL) and kept in moist, dark conditions for 24 h to promote infection. Non-inoculated plants were used as controls. Samples were collected from wheat leaves at 0, 24 and 48 hours post-inoculation and stored at -80°C.

### 2.8 RNA extraction and real-time quantitative PCR

Total RNA was extracted from the wheat leaves using TRIzol reagent (Invitrogen, Gaithersburg, MD, USA). RNA quality was examined using NanoDrop one spectrophotometer and electrophoresis. Complementary DNA was synthesized using HiScript Reverse Transcriptase (Vazyme, Nanjing, China) according to the manufacturer’s protocol. ChamQ SYBR qPCR Master Mix (Vazyme, Nanjing, China) was used to perform the RT-qPCR in a 20 µL reaction. The thermal profiles used are as follows: stage 1: 95°C for 3 min; stage 2: 95°C for 10 s and 60°C for 30 s for 40 cycles; and stage 3: 95°C for 15 s, 60°C for 1 min, and 95°C for 15 s. Three biological replicates were processed for each sample, and the relative expression levels were calculated by the 2-ΔΔCT method, with the ADP-ribosylation factor Ta2291 serving as the internal reference control (Huang et al., 2021).

### 2.9 Protein-RNA complex structure prediction using AlphaFold server

To investigate the spatial interaction mechanisms underlying the regulatory relationships between differentially expressed lincRNAs and their adjacent target genes, we utilized AlphaFold Server (https://alphafoldserver.com/) for protein–RNA complex structure prediction. The nucleotide sequence of the candidate lincRNA MSTRG.56980 and the amino acid sequence of the target protein TraesCS6B03G0179100 were retrieved from the assembled transcriptome data and the Ensembl Plants database, respectively, and were submitted directly to the server for model building with all parameters retained at their default settings.

The predicted complex structure was exported in PDB format and subjected to three-dimensional visualization analysis using PyMOL (v2.5). For clarity in presenting the interaction interface, the target protein and the lincRNA were rendered in blue and green, respectively. For the predicted complex with pTM > 0.5 and ipTM > 0.8, For the predicted complex with pTM > 0.5 and ipTM > 0.8, nucleotide residues of the lincRNA and amino acid residues of the target protein with interatomic distances of less than 5.0 Å were defined as the core binding interface and highlighted in red. This distance threshold effectively identified potential intermolecular contact regions and facilitated the quantitative estimation of the binding interface area.

## 3 Results

### 3.1 Systematic identification and characterization of wheat lincRNAs under *Pst* infection

A comprehensive computational pipeline was applied to 197,328 assembled transcripts to generate a high-confidence catalog of wheat lincRNAs responsive to *Pst* infection (**Figure 1A**). Initial transcript classification using Gffcompare identified 30,681 intergenic transcripts (class code “u”), representing 15.55% of all assembled transcripts (**Figure 1B**). Known protein-coding isoforms (class code “=“), which accounted for the majority of transcripts, were excluded from further analysis. Subsequent structural filtering removed single-exon transcripts and those shorter than 200 bp and reduce the dataset to 25,688 candidates.

**Figure 1:**
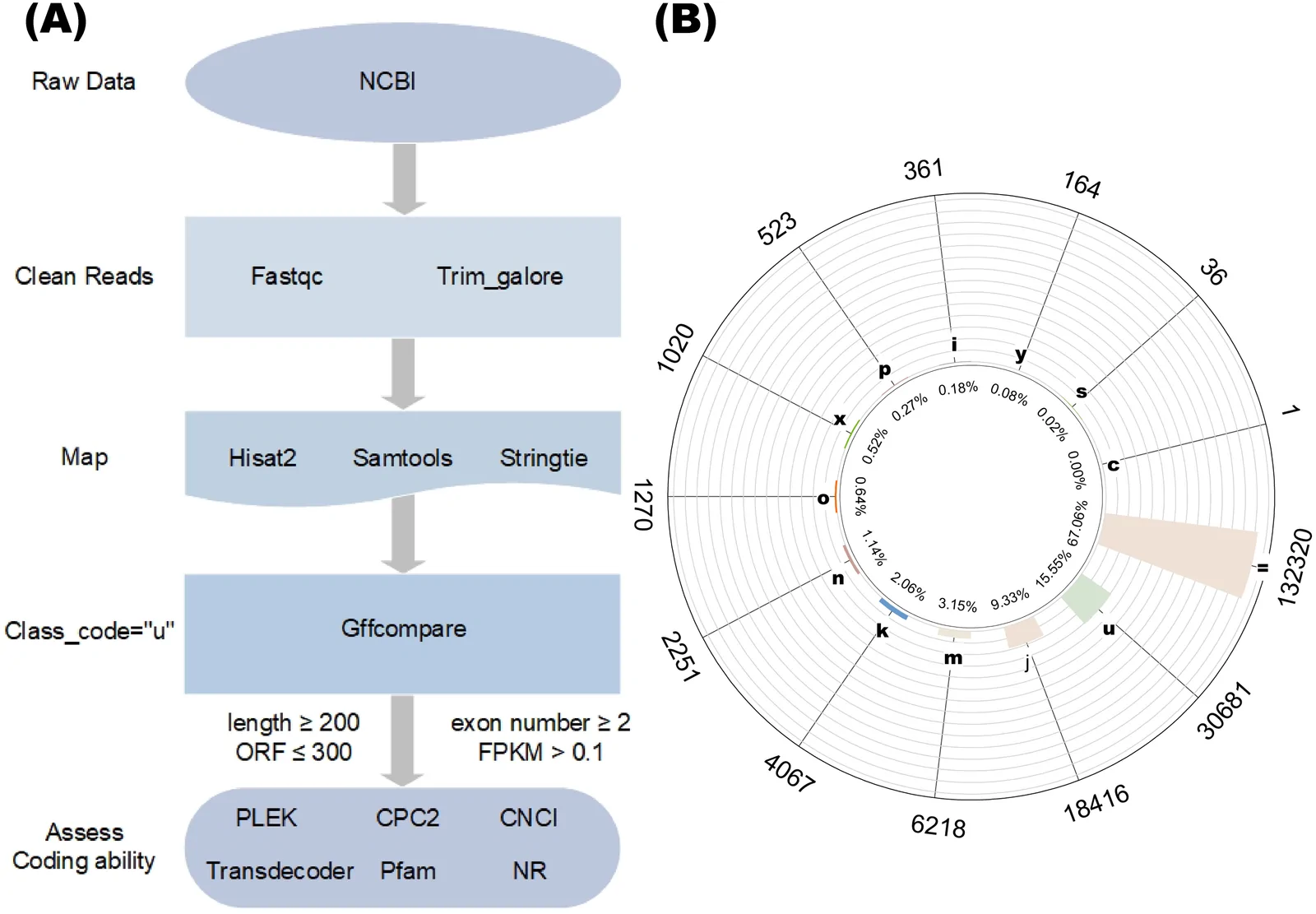
Identification and characterization of lincRNAs. **(A)** A flowchart for lincRNA identification; **(B)** The class_code represents the position and quantity of transcripts relative to the reference genome.= exact match to known transcript; u/x/i intergenic/antisense/intronic lncRNAs; c/k/j/m/n/o novel isoforms of known genes;/x/p/s potential artifacts.

PLEK, CPC2, and CNCI tools were applied to eliminate protein-coding transcripts, yielding 6,559 high-confidence non-coding candidates. These sequences were further screened through six-frame translation followed by domain and homology filtering. HMMER searches against the Pfam database and BLASTX searches against the NCBI NR protein database removed 1,823 transcripts with significant similarity to known protein domains or proteins.

Expression support was then assessed, retaining only transcripts with FPKM > 0.1 in at least one biological replicate. This final filtering step produced 2,736 expressed lincRNAs. Differential expression analysis identified 515 lincRNAs significantly regulated during *Pst* infection. Among these, 34 lincRNAs were located within 100 kb of differentially expressed genes (DEG) encoding pathogenesis-related proteins or transporters, and he final dataset represents 0.26% of all initial transcripts.

### 3.2 Characterization of wheat long intergenic ncRNAs

Comparative analysis of structural features revealed clear distinctions between wheat lincRNAs and mRNAs. Using the 515 differentially expressed (DE) lincRNAs and 30,681 annotated mRNAs, exon number, transcript length, and expression patterns were examined to assess whether wheat lincRNAs exhibit characteristics typical of plant non-coding transcripts. Both lincRNAs and mRNAs showed an inverse relationship between exon number and transcript abundance, but lincRNAs were strongly enriched for low-exon structures, consistent with their streamlined architecture (**Figure 2A**). Transcript length distributions demonstrated that lincRNAs are substantially shorter than mRNAs. Expression analysis revealed that lincRNAs accumulate at lower levels than mRNAs (**Figure 2B**).

**Figure 2:**
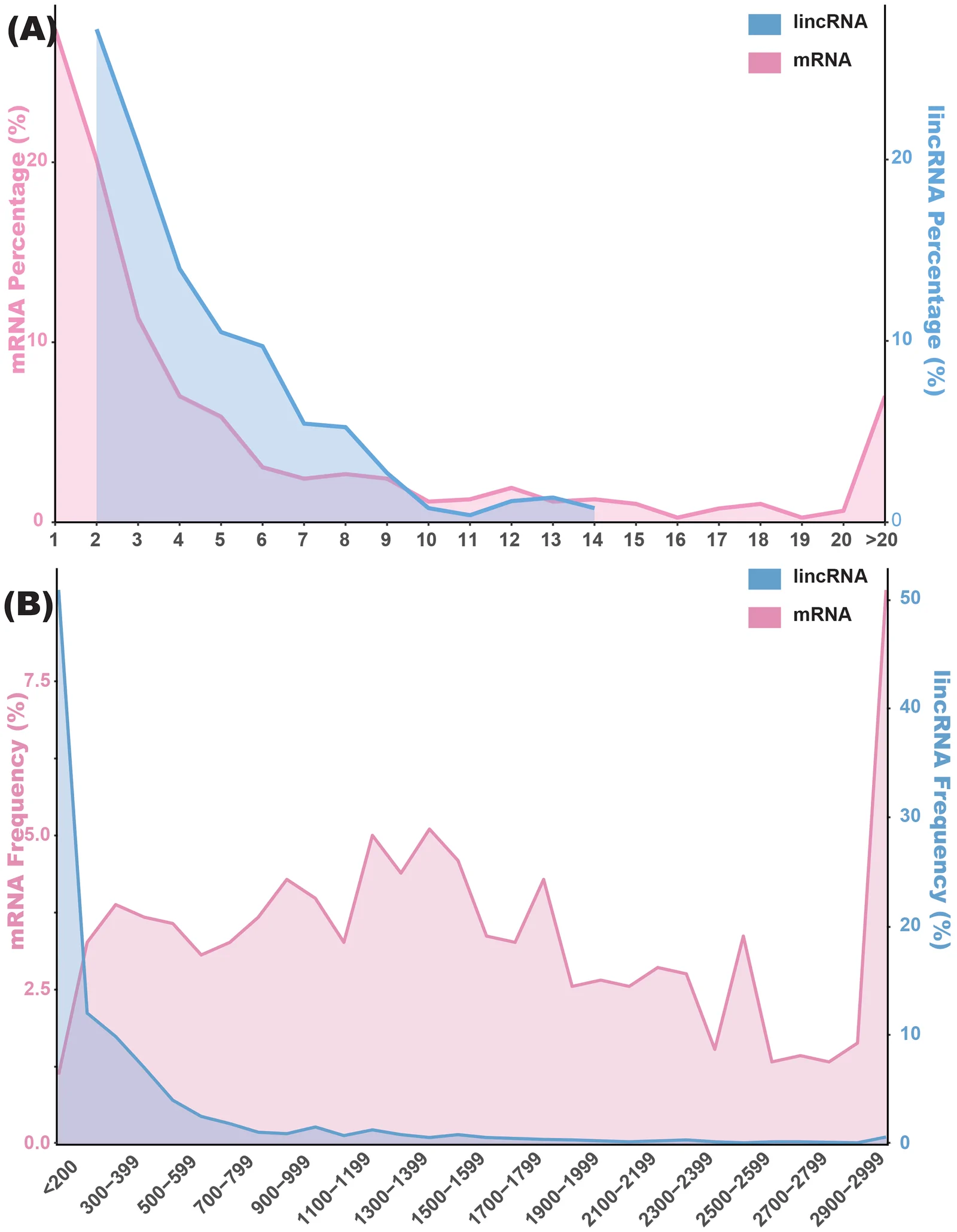
Characterization of wheat lincRNAs. **(A)** Distribution of exon numbers in lincRNAs and mRNAs; **(B)** Distribution of transcript lengths in lincRNAs and mRNAs.

LincRNA expression exhibited substantial variability following *Pst* inoculation (**Figures 3A–F**). At 6 h post-infection, 12 lincRNAs were upregulated and 32 were downregulated (**Figure 3G**). By 12 h, the number of upregulated lincRNAs increased to 56, while 18 were downregulated. A pronounced transcriptional response was observed at 24 h, with 38 upregulated and 221 downregulated lincRNAs. Pairwise comparisons between infection stages further showed dynamic regulatory changes. Between 6-12 h, 55 upregulated and 15 downregulated lincRNAs were identified. Between 6-24 h, 19 upregulated and 11 downregulated lincRNAs were detected. The 12-24 h interval showed the strongest shift, with 36 upregulated and 317 downregulated lincRNAs. These comparisons showed dynamic changes in lincRNA expression across infection stages.

**Figure 3:**
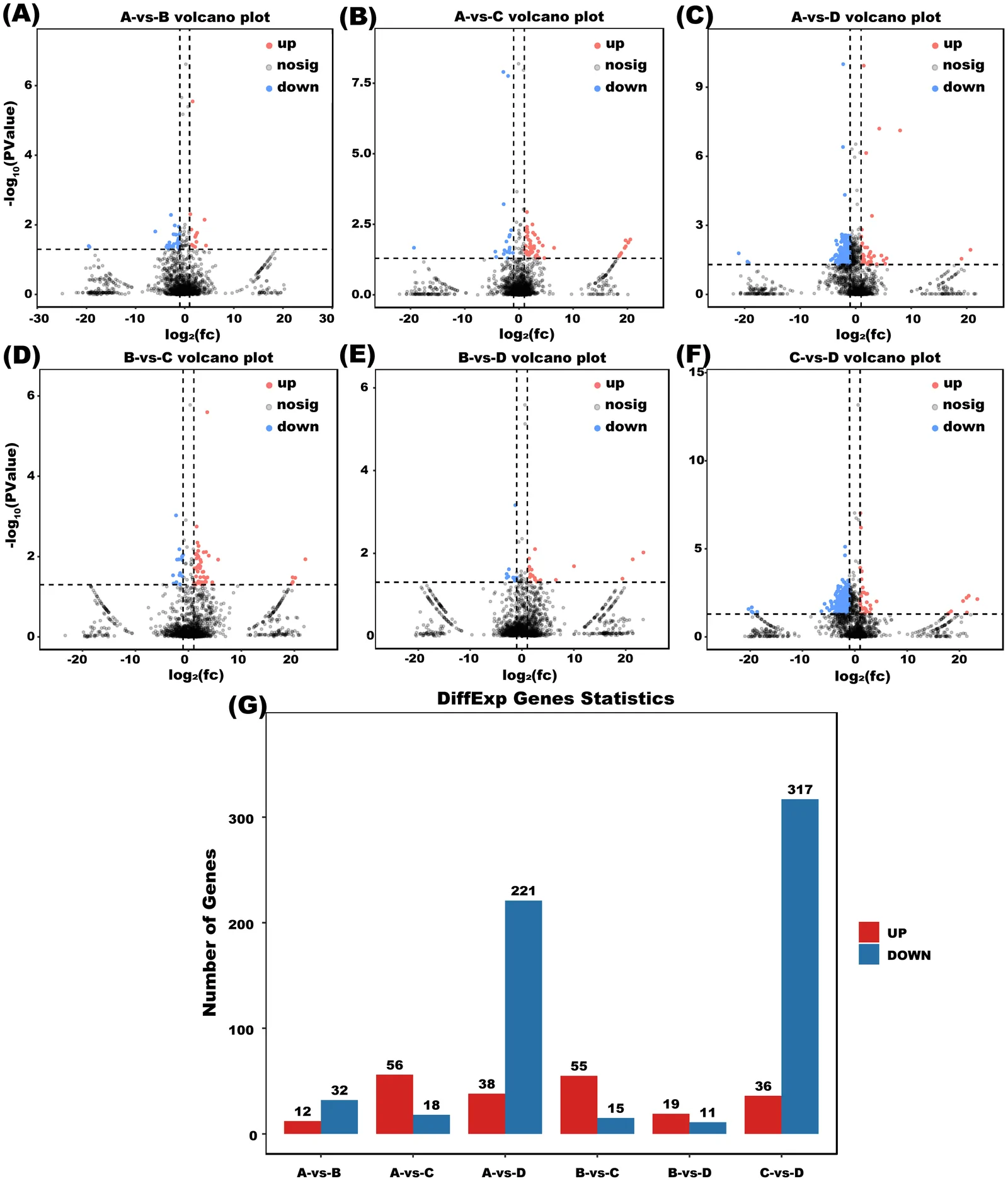
Differential expression of lincRNAs in wheat during *Pst* infection. **(A–F)** Volcano plots of pairwise comparisons between groups **(A)** 0 H; **(B)** 24 H; **(C)** 48 H; **(D)** 120 H; **(G)** Summary bar plot of DEG numbers. UP/DOWN: Genes with increased/decreased expression.

### 3.3 Functional annotation and pathway enrichment analysis of lincRNA-associated target genes

Functional annotation of genes located near differentially expressed lincRNAs revealed enrichment for immune-related and stress-responsive processes during *Pst* infection. A total of 834 adjacent mRNAs were subjected to GO and KEGG enrichment analyses. In the Biological Process (BP) category, enriched terms were primarily associated with cellular processes and metabolic processes (**Figure 4A**). Cellular Component (CC) terms were enriched for cellular metabolic entities and protein-containing complexes, while Molecular Function (MF) terms were dominated by binding and catalytic activities. GO enrichment analysis identified significant BP terms related to the extracellular region and organelle small ribosomal subunits (**Figures 4B, E**). For CC, significantly enriched terms included S-formylglutathione hydrolase activity and rRNA binding (**Figures 4C, F**). The most enriched MF terms were associated with actin filament bundle assembly and actin filament bundle organization (**Figures 4D, G**), processes often linked to cytoskeletal remodeling during pathogen attack..

**Figure 4:**
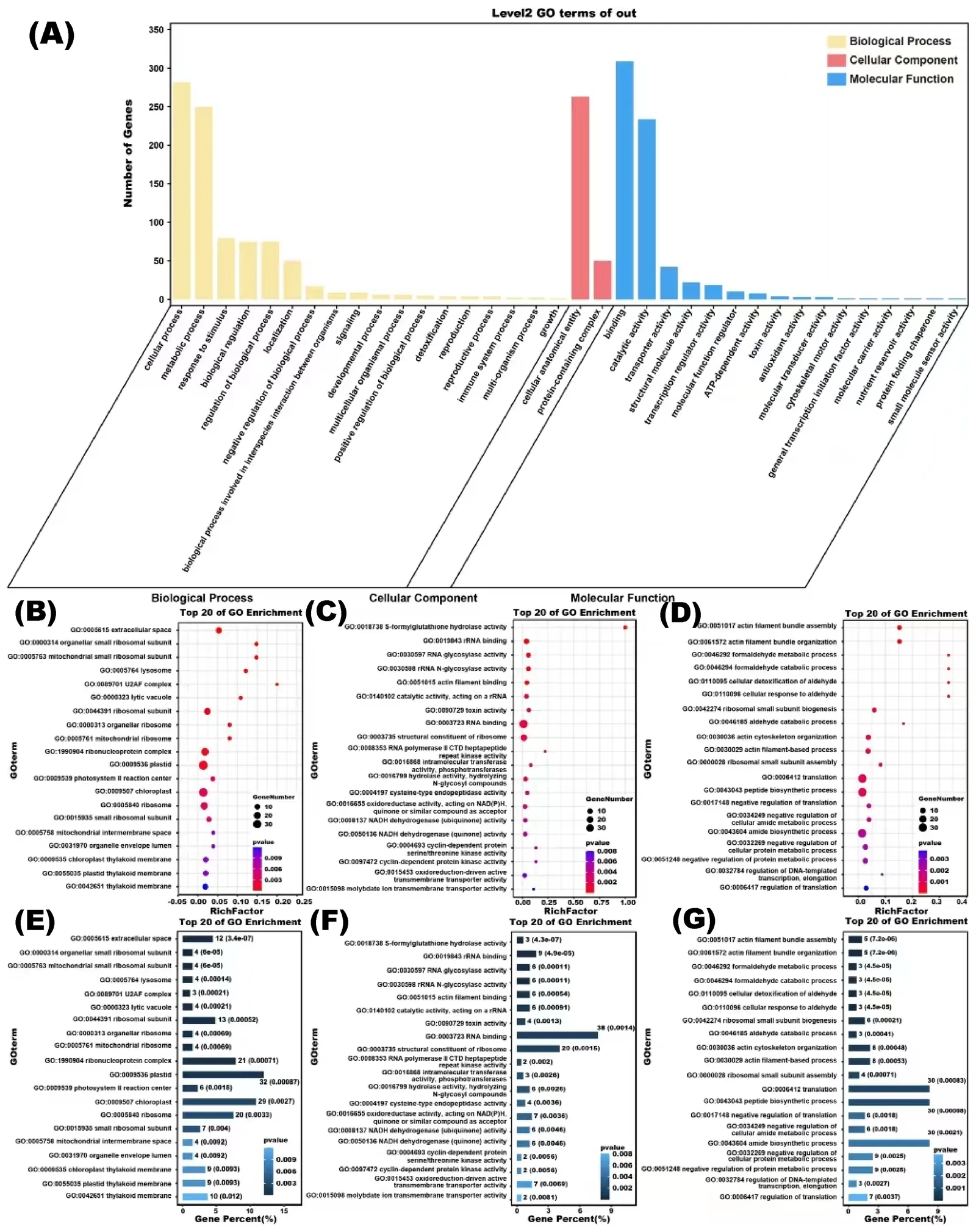
GO term enrichment analysis of 515 differentially expressed lincRNA targets. **(A)** Statistical bar chart of GO secondary classifications. The number of genes utilized for enrichment is calculated for each GO category. The horizontal axis represents the three GO ontology categories. The vertical axis indicates the number of genes in each category. **(B–D)** Go enriched bubble map. Only enriched GO term (P-value < 0.05). The top 20 of BP, MF and CC are shown. The circle size represented the gene number. Red to purple represents the low to high P-value. **(E–G)** GO Enrichment Analysis Bar Chart. The abscissa is the proportion of the number of genes, and the ordinate is the GO Term and the details of each GO number. The different color depths in the figure represent different gene numbers, and the color gradually decreases from dark to light. From left to right, it corresponds to the specific classification of BP, CC, and MF.

KEGG pathway classification indicated that global and overview metabolic pathways were prominently represented (**Figure 5A**). Within the major KEGG categories, enriched pathways included translation, signal transduction, transport and catabolism, and environmental adaptation (**Figure 5B**). KEGG enrichment analysis identified ribosome, photosynthesis, and D-amino acid metabolism as the most significantly enriched pathways, as shown in the top 15 enriched pathway bubble plot (**Figure 5C**), in which circle size indicates gene count and color intensity indicates -log10(Pvalue).

**Figure 5:**
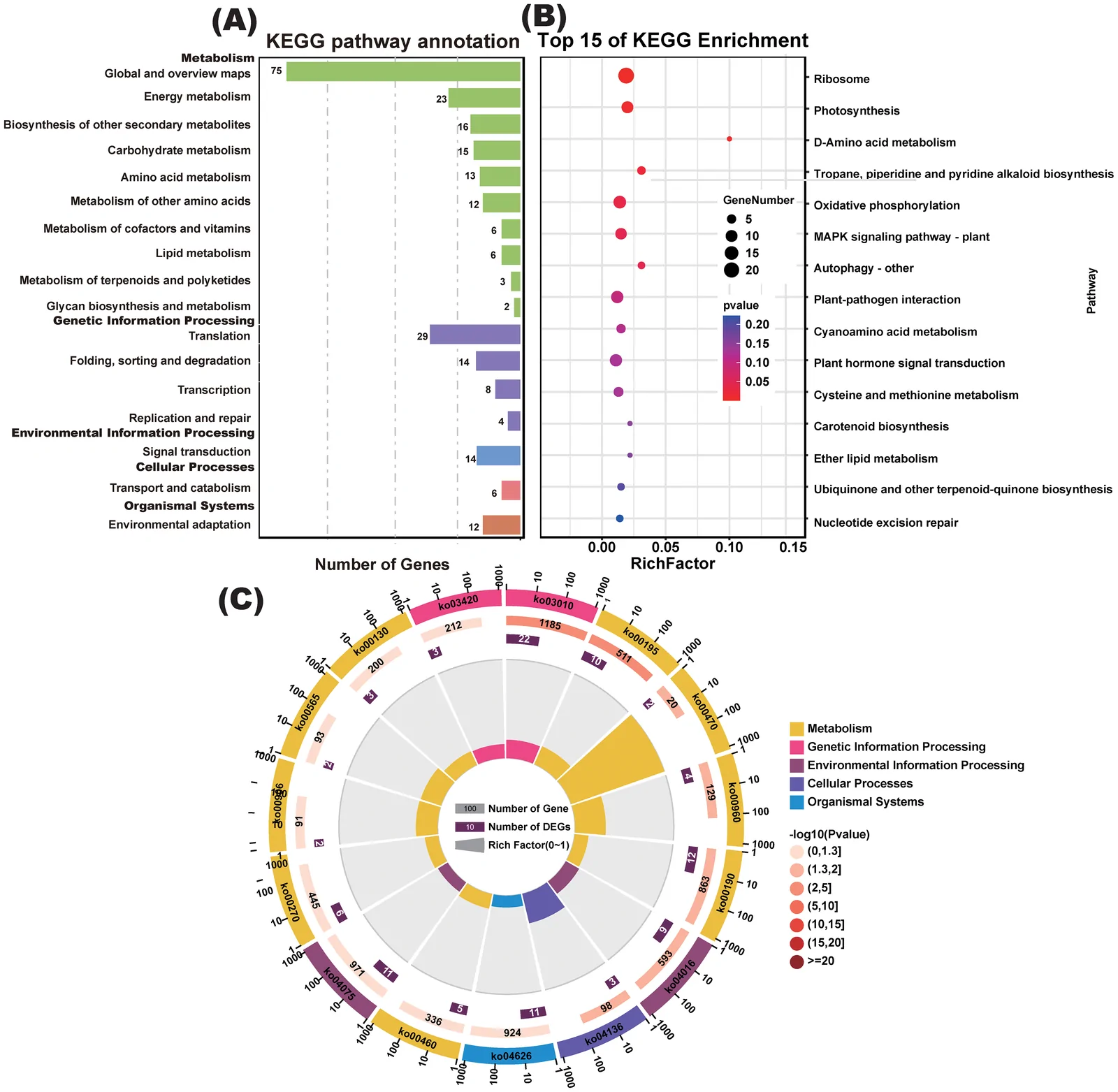
KEGG pathway enrichment of DE-lincRNA target genes. **(A)** Circular plot of enriched pathways. Outer ring: Pathway names; inner color blocks: KEGG Level-1 categories. **(B)** Annotation statistics for Level-2 subcategories. **(C)** Bubble plot of top 15 enriched pathways (circle size: gene count; color: -log_10_ (P), red=most significant).

### 3.4 Expression analysis of lincRNA-mRNA co-expression under *Pst* Infection

To evaluate whether DE-lincRNAs exhibit coordinated regulation with nearby mRNAs during *Pst* infection, expression patterns of selected lincRNA-mRNA pairs were examined using qPCR. The expression levels of MSTRG.18458-Ta2B1, MSTRG.18458-Ta2B2, and MSTRG.18458-Ta2B3 were downregulated throughout the treatment periods compared to the control (**Figures 6A–C**). Expression of levels of MSTRG.34447-Ta3A1 and MSTRG.34447-Ta1D2 reduced at 24 h before increased after 48 h treatment (**Figures 6D, E**). Expression levels of Ta3D1 and MSTRG.56980-Ta6B1 increased with increasing treatment periods (**Figures 6F, G**), while expression levels of Ta7B1 increased and peaked after 24 h before downregulating (**Figure 6H**). Functional annotation identified Ta6B1 as an RGA1-type disease resistance protein. Conserved domain analysis identified an Rx N-terminal domain (PF18052) in TraesCS6B03G0179100, a domain characteristic of plant NLR resistance proteins. Although an automated KEGG annotation assigned this gene to K15078 (SLX1 endonuclease), this assignment is inconsistent with the presence of PF18052 and lacks structural support. Therefore, the K15078 annotation was considered a false-positive KEGG assignment.

**Figure 6:**
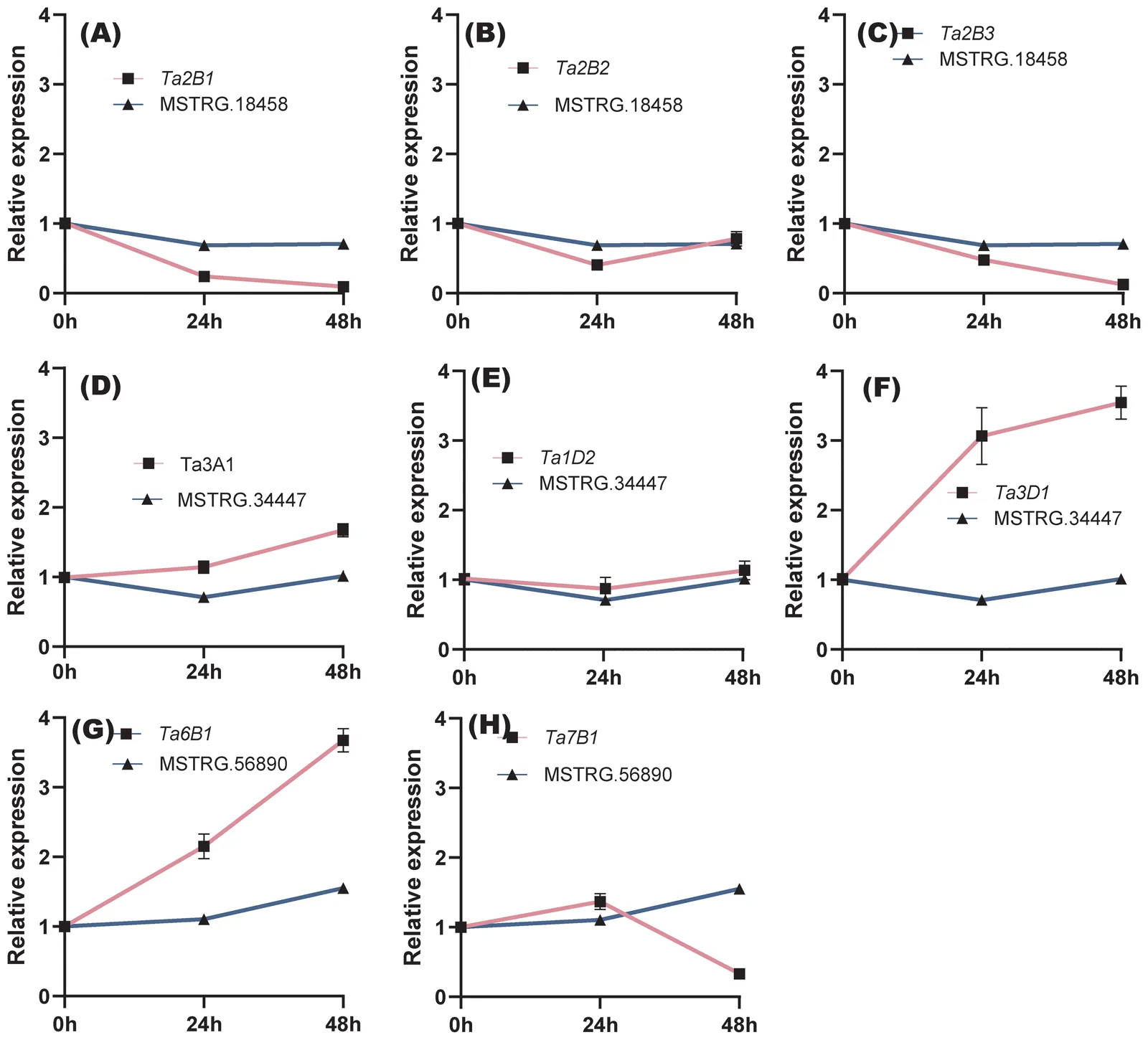
The figure shows the expression pattern of lincRNAs.Data are from three biological replicates, and error bars indicate SE. **(A)** MSTRG.18458-Ta2B1. **(B)** MSTRG.18458-Ta2B2. **(C)** MSTRG.18458-Ta2B3. **(D)** MSTRG.34447-Ta3A1. **(E)** MSTRG.34447-Ta1D2. **(F)** MSTRG.34447-Ta3D1. **(G)** MSTRG.56980-Ta6B1. **(H)** MSTRG.56980-Ta7B1.

### 3.5 Prediction of lincRNA-miRNA-mRNA ceRNA networks

To investigate whether the identified DE-lincRNAs potentially function as miRNA sponges, we performed a genome-wide prediction using the psRNATarget webserver with an Expectation score threshold of ≤ 4.5. A total of 119 mature wheat miRNAs (retrieved from miRBase) were screened against the 515 DE-lincRNAs, and 108 miRNAs were found to have potential targeting interactions with these lincRNAs. These 108 miRNAs were then searched against wheat protein-coding gene transcripts, yielding 377 predicted target genes. Network analysis revealed that a single miRNA could target multiple lincRNAs, and conversely, individual lincRNAs were associated with multiple miRNAs, indicating a complex multi-to-multi regulatory architecture. Ultimately, 65 DE-lincRNAs were computationally predicted as potential eTMs for 21 conserved miRNAs (**Figure 7**). Network analysis showed that a single miRNA could target multiple lincRNAs, and individual lincRNAs were associated with multiple miRNAs, forming a multi-to-multi interaction architecture.

**Figure 7:**
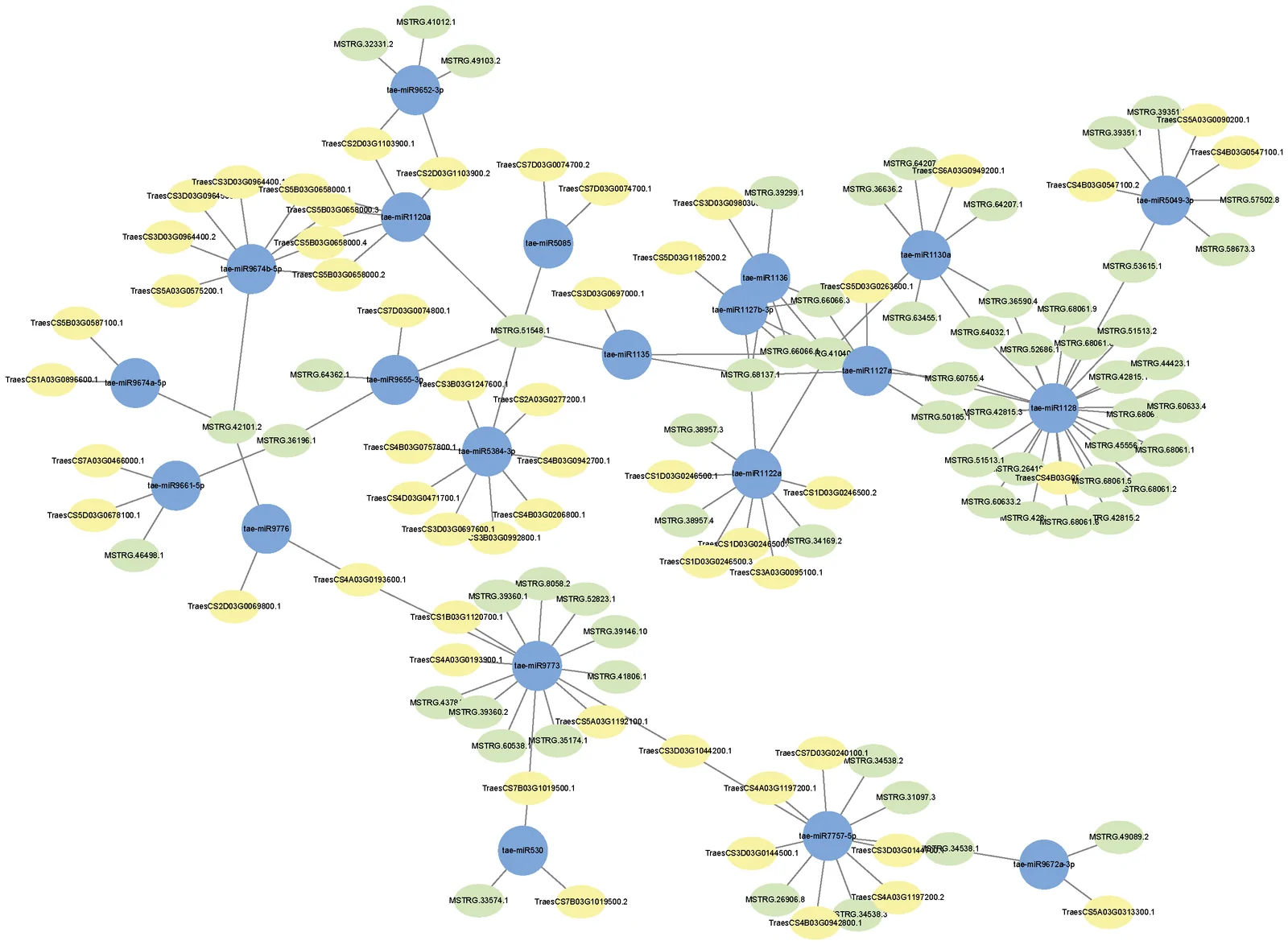
Predicted lincRNA-miRNA-mRNA regulatory network in wheat during *Pst* infection.Green circles, differentially expressed lincRNAs; blue circles, conserved miRNAs; yellow circles, predicted target mRNAs.

### 3.6 Spatial interaction interface analysis between the candidate lincRNA and its target protein

To further explore the potential regulatory relationship between the lincRNA and its target gene at the three-dimensional structural level, we performed protein-RNA complex structure prediction for the co-expressed lincRNA MSTRG.56980 and the protein TraesCS6B03G0179100 encoded by its adjacent defense-related gene. AlphaFold-based protein-RNA structure prediction generated a model in which MSTRG.56980 was predicted to interact with TraesCS6B03G0179100 (**Figure 8**). The predicted interface included local base-pairing and stacking interactions between the lincRNA and amino acid residues of the target protein.

**Figure 8:**
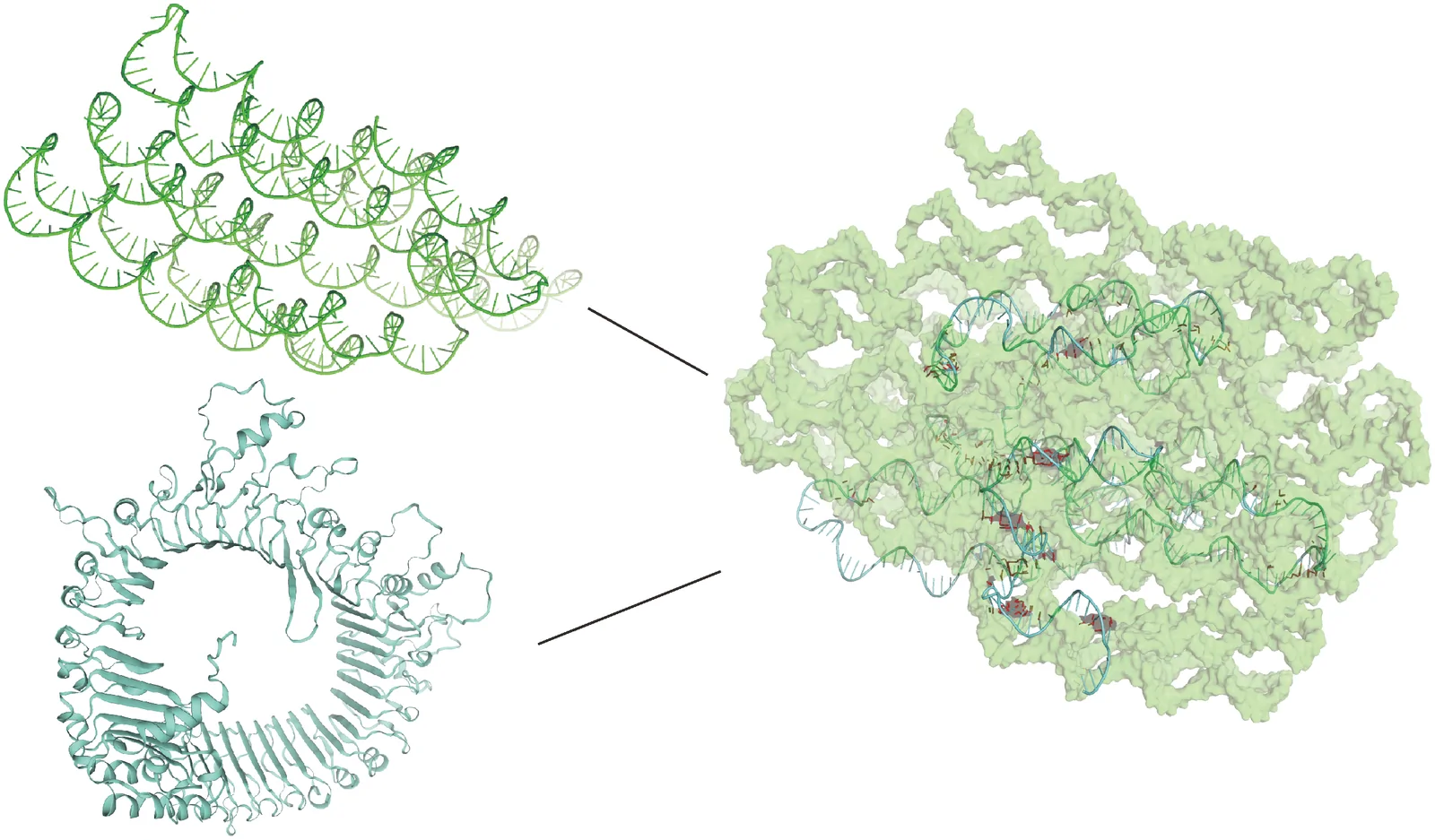
Predicted protein-RNA complex structure between MSTRG.56980 and TraesCS6B03G0179100. The lincRNA is shown in green, the target protein in blue, and the binding interface is highlighted in red.

## 4 Discussion

LincRNAs have emerged as key regulatory molecules in plant stress responses, influencing transcriptional activation, chromatin remodeling, and post-transcriptional regulation across diverse species (Huang et al., 2023). Increasing evidence shows that lincRNAs participate in immune signaling, hormone-mediated defense, and pathogen recognition pathways, functioning as both positive and negative regulators of plant immunity (Wang and Folimonova, 2023; Bhat et al., 2025). In *Arabidopsis*, ELENA1 enhances resistance to *Pseudomonas syringae* by recruiting mediator subunits to activate PR1 transcription (Mach, 2017), while the lincRNA DRIR modulates drought and salt tolerance through ABA-dependent signaling. Similar regulatory roles have been reported in rice, where ALEX1 activates jasmonate-mediated immunity (Yu et al., 2020), and in maize, where stress-responsive lincRNAs derived from transposable elements function as regulatory hubs coordinating abiotic and biotic stress pathways (Lv et al., 2019). Despite these advances, the functional landscape of lincRNAs in wheat remains poorly defined, largely due to the complexity and redundancy of its allohexaploid genome, which complicates transcript annotation and functional inference (Liu et al., 2026). Several studies have reported that wheat lncRNAs respond to fungal pathogens, including powdery mildew. Fusarium head blight, and stripe rust have focused on global expression patterns rather than mechanistic characterization (Duan et al., 2020). However, only a few lincRNAs have been experimentally linked to wheat immunity (Su et al., 2025). This study generated a catalog of wheat lincRNAs responsive to stripe rust infection.

The structural and expression characteristics observed in wheat lincRNAs in this study are consistent with the broader biological patterns described for lncRNAs. Wheat lincRNAs were generally shorter, contained fewer exons, and exhibited lower expression levels than protein-coding genes, reflecting the canonical features of plant lncRNAs as low-abundance, structurally simple regulatory molecules (Chen and Zhu, 2022). These characteristics align with the definition of lncRNAs as transcripts longer than 200 nucleotides with limited coding potential and often restricted expression patterns (Yamada, 2017). The observation that wheat lincRNAs displayed greater expression variability across cultivars than mRNAs suggests that these transcripts are potentially highly sensitive to genetic background and may contribute to genotype-specific immune responses. This is consistent with findings that plant lncRNAs evolve rapidly, show limited sequence conservation, and often function in a species- or genotype-dependent manner (Lanctot, 2023). The compact structure and low exon number of wheat lincRNAs support the idea that they act as regulatory elements rather than structural RNAs, consistent with the understanding that plant lncRNAs modulate gene expression through interactions with chromatin, transcription factors, or other RNAs (Gil and Ulitsky, 2020). Studies have reported that lincRNAs can influence the expression of nearby mRNAs through the local propagation of similar chromatin configurations (Greenstein et al., 2018). These results suggest that wheat lincRNAs potentially function as dynamic regulatory components of the immune transcriptome that may contribute to cultivar-specific defense strategies against *Pst* infection (Bedre et al., 2024).

The expression of wheat lincRNAs during *Pst* infection in this study reveal a highly coordinated and phased transcriptional response. Early infection stages were marked by moderate induction of lincRNAs, suggesting potential rapid activation of pathogen recognition and signaling pathways. However, a pronounced shift toward downregulation was observed at the late stage. This pattern aligns with the emerging understanding that plant lncRNAs participate in both the initiation and attenuation of immune responses (Seo et al., 2026). LncRNAs can modulate chromatin accessibility, transcriptional activation, and RNA stability during pathogen challenge (Fernandes et al., 2019). The downregulation at later infection stages may indicate a feedback mechanism that prevent excessive immune activation, a phenomenon reported in other plants where lncRNAs fine-tune defense responses to balance immunity and growth (Cui, 2023). A study reported that lincRNAs modulate proximal mRNAs expression and tolerance to *Candidatus Liberibacter* spp. in potatoes. LincRNAs were found to regulate plant defense hormone pathways (Liu et al., 2022). In *Arabidopsis*, 20 lincRNAs were found to be differentially expressed upon infection with the soil-borne fungal pathogen Fusarium oxysporum. Among these, several were shown to modulate disease development, potentially through their regulatory effects on adjacent genes (Zhu et al., 2014). LincRNA *MSTRG.55194* enhances resistance to *Fusarium* crown rot by binding to target genes and regulating defense-related gene expression in wheat (Ma et al., 2025). In wheat, a study identified 254 DE-lincRNAs under powdery mildew treatmeent and 52 lincRNAs after stripe rust infection. Several of these lncRNAs were predicted to act as targets or target mimics of miRNAs, potentially regulating wheat resistance to both pathogens through miRNA-mediated pathways. Also, a transcriptome analysis identified 1,238 expressed lincRNAs, of which 548 were differentially expressed at various time points after *F. graminearum* infection (Su et al., 2025).The dynamic expression patterns of wheat lincRNAs across infection stages suggest that they may contribute to stage-specific regulatory processes during wheat-*Pst* interactions. Early induction may be associated with pathogen recognition and signaling, whereas the pronounced downregulation at later stages may reflect a feedback mechanism that prevents excessive immune activation. Similar phased responses have been reported for plant lncRNAs during pathogen infection.

Functional enrichment of mRNAs located near DE- lincRNAs reveals that these transcripts occupy genomic regions deeply embedded in wheat immune regulation. Adjacent genes were associated with several defense-related pathways such as MAPK signaling pathway, photosynthesis, and plant-pathogen interaction. MAPK cascades are significant signaling pathways positioned downstream of receptors that detect both internal stimuli such as peptide ligands and damage-associated molecular patterns, and external signals, including pathogen- or microbe-associated molecular patterns, and environmental conditions (Zhang and Zhang, 2022). MAPK activation during effector-triggered immunity triggers inhibition of photosynthesis, increased ROS generation within chloroplasts, and promotion of programmed cell death (Jianbin et al., 2018). Photorespiration connects photosynthetic carbon assimilation to other pathways, including nitrogen and sulfur metabolism and the tricarboxylic acid cycle (Abadie and Tcherkez, 2019). This functional integration is essential for the allocation of metabolic resources and energy required to activate defense mechanisms against pathogens. Meanwhile, environmental factors such as temperature and CO_2_ levels regulate photorespiration and affect both pathogens and plant–pathogen interactions (Stefan and Martin, 2020; Lahlali et al., 2024). Plants perceive pathogens through membrane receptors that recognize pathogen-associated molecular patterns (PAMPs) or intracellular receptors that detect pathogen effectors. This triggers complex signaling cascades, including MAPK pathways, calcium fluxes, and hormonal networks involving salicylic acid, jasmonic acid, and ethylene. These signals activate transcriptional reprogramming, leading to defense outputs such as ROS production, cell wall reinforcement, antimicrobial compound synthesis, and programmed cell death (Ding et al., 2022; Simas et al., 2025). The enrichment of actin filament bundle assembly and organization suggests potential cytoskeletal remodeling during pathogen attack. Similarly, the enrichment of ribosome, photosynthesis, and D-amino acid metabolism pathways may indicate extensive metabolic and translational reprogramming associated with *Pst* infection.

Beyond cis-regulation, our lincRNA-miRNA-mRNA network computationally predicted 65 DE-lincRNAs as potential endogenous target mimics for 21 conserved miRNAs. The multi-to-multi architecture suggests that wheat lincRNAs may act as integrators of miRNA-mediated signals, potentially coordinating multiple defense pathways. This provides preliminary computational evidence for a putative ceRNA mechanism during wheat–*Pst* interactions.

Furthermore, AlphaFold-based protein-RNA structure prediction suggested a potential complex between MSTRG.56980 and TraesCS6B03G0179100, providing computational evidence for a possible physical association. The predicted binding mode raises the possibility that MSTRG.56980 may associate with the target protein and modulate its function or interfere with interactions between the target protein and other immune components.

The Ta6B1-MSTRG.56980 pair exemplifies this regulatory potential: Ta6B1 encodes a disease-related protein, and its coordinated induction with the adjacent lincRNA suggests a cis-regulatory module where lincRNAs potentially modulate NLR gene activation and pathogen-triggered signaling (Devadhasan et al., 2025). Protein-coding genes whose expression is altered by lincRNA loss of function are its targets. Upregulation defines the lincRNA as a repressor, while downregulation defines it as an activator. Since then, several cis-regulatory cis-lncRNAs have been described (Rom et al., 2019). Other lncRNAs, however, lack an obvious positional link to their targets and are termed trans-lncRNAs (Leucci et al., 2016). This cis/trans distinction forms a core framework for understanding regulatory lncRNAs (Bao et al., 2025). Cis-acting lncRNAs account for a substantial share of functional lncRNAs. Their regulatory activity depends on the genomic location of their transcription relative to target genes, which can be nearby or farther along the linear genome. These findings suggest that wheat lincRNAs potentially act as an integral components of several immune regulatory systems (Wu et al., 2017). Further research is necessary to fully characterize DE-lincRNAs in wheat during stripe rust infection to understand their regulatory roles in wheat-pathogen interactions. The coordinated expression of Ta6B1 and MSTRG.56980 suggests a putative cis-regulatory association, in which the lincRNA may potentially modulate NLR gene activation and pathogen-triggered signaling. However, this hypothesis requires functional validation. Beyond lincRNA-mediated regulation, RNA-based strategies such as dsRNA expression have also been explored for crop protection against pests (Zhang et al., 2025), although their application to fungal pathogens and lincRNA biology remains to be explored.

## 5 Conclusions

This study provides a systematic identification of lincRNAs in wheat responding to stripe rust infection. A total of 2,736 expressed lincRNAs were identified, among which 515 were differentially expressed during infection, with a pronounced shift toward downregulation at later time points. Structural and expression characteristics of wheat lincRNAs align with conserved features of plant non-coding transcripts, including shorter length, fewer exons, and lower abundance compared to protein-coding genes. Functional enrichment of lincRNA-associated genomic regions revealed their proximity to genes involved in immune signaling, photosynthesis, and metabolic reprogramming. Coordinated expression patterns between lincRNAs and adjacent defense-related mRNAs supported the potential involvement of lincRNA-mRNA modules in wheat–pathogen interactions. Furthermore, integrative network analysis computationally predicted 65 DE-lincRNAs as potential endogenous target mimics for 21 conserved miRNAs, suggesting a putative ceRNA regulatory mechanism, while protein–RNA structure prediction revealed a stable complex between the lincRNA MSTRG.56980 and the RGA1-type resistance protein TraesCS6B03G0179100, providing structural insights into potential physical interactions. These findings provide a candidate resource and generate hypotheses for future functional characterization of lincRNAs in wheat stripe rust resistance and disease resistance breeding (Jin et al., 2024; Wang et al., 2024).

## Data Availability

Publicly available datasets were analyzed in this study. This data can be found in the NCBI SRA (https://www.ncbi.nlm.nih.gov/sra) under BioProject accession PRJNA975982.
